# Sea-level rise projections for Sweden based on the new IPCC special report: The ocean and cryosphere in a changing climate

**DOI:** 10.1007/s13280-019-01313-8

**Published:** 2020-01-28

**Authors:** Magnus Hieronymus, Ola Kalén

**Affiliations:** Swedish Meteorological and Hydrological Institute, Norrköping, Sweden

**Keywords:** Extreme sea levels, Sea-level rise, SROCC, Sweden

## Abstract

New sea-level rise projections for Sweden are presented. Compared to earlier projections, we have here, more carefully, taken regional variations in sea-level rise into consideration. The better treatment of regional variations leads to lower sea-level rise projections for Sweden. However, recent research has also shown that Antarctic ice loss, in high emission scenarios, could be greater than what was believed earlier. Taking also this into account, we find a near cancellation between the increased Antarctic contribution and the decrease owing to the better treatment of spatial inhomogeneities. Sweden’s sensitivity to melt from Antarctica and Greenland is also estimated using a new set of sea-level fingerprint kernels, and the sensitivity to melt from Greenland is found to be weak. To illustrate the influence mean sea-level rise has on extreme sea levels, it is also shown how the return period of sea-level extremes changes as a function of time owing to mean sea-level rise in the different projections.

**Electronic supplementary material:**

The online version of this article (doi:10.1007/s13280-019-01313-8) contains supplementary material, which is available to authorized users.

## Introduction

The primary aim with this paper is to introduce updated sea-level rise projections for Sweden that can be used, for example, to support coastal spatial planning. The projections currently used for most such planning in Sweden (Nerheim et al. 2018) were released, as part of a technical report written in Swedish, by the Swedish Meteorological and Hydrological Institute (SMHI) and are based on the Intergovernmental Panel on Climate Change: Fifth Assessment Report (IPCC: AR5) (Church et al. 2013). These projections use the simplification that the mean sea level at the Swedish coast is expected to rise like the global mean sea level, corrected for the local glacial isostatic adjustment (GIA). GIA is also called post-glacial rebound and it is considerable throughout large parts of the Scandinavian Peninsula. The simplification of neglecting spatial inhomogeneities apart from in GIA is, however, unnecessary since the spatial patterns of sea-level rise owing to land ice melt, ground water changes and ocean-atmosphere dynamics are also estimated in AR5. Moreover, several works that emphasize the spatial variability of sea-level rise on global and more regional scales for future climate scenarios have been published in scientific journals since the release of AR5 (see, e.g. Johansson et al. 2014; Kopp et al. 2014; Grinsted et al. 2015; Jevrejeva et al. 2019).

Using individual scientific journal articles for coastal spatial planning is, however, impractical owing both to the high production rate of such articles and also to the fact that all published scientific findings do not stand the test of time. For smaller nations, it is, however, not feasible to produce their own summaries of current knowledge at regular intervals, and the assessments offered by the IPCC are consequently most often the underlying basis used for planning. IPCC’s material is then often translated and complemented with, for example, local downscaling efforts. In here, we will present new sea-level projections for Sweden based on the new IPCC special report: The Ocean and Cryosphere in a Changing Climate (SROCC) (Oppenheimer et al. 2019), which was released on September 25, 2019. Moreover, we will take into account the full spatial variability of sea-level rise as described in SROCC. That is, these projections take into account regional sea-level changes owing to GIA, land ice melt, ground water changes, steric effects and changes in sea-level pressure and winds.

The new projections are given for the representative concentration pathways (RCPs) 2.6, 4.5 and 8.5. These different climate scenarios are labelled after their respective radiative forcing in the year 2100, which consequently is 2.6, 4.5 and 8.5 ${\text{Wm}}^{-2}$. In RCP2.6, emissions peak around 2020, while in RCP8.5 they continue to grow throughout the century (van Vuuren et al. 2011). The *likely* range of the temperature increase for 2081–2100 relative to 1986–2005 is 0.3–1.7 °C under RCP2.6 and 2.6–4.8 °C under RCP8.5 (IPCC 2013). The SROCC and AR5 projections are based on the same suite of coupled climate model experiments from the Coupled Model Intercomparison Project Phase 5 (CMIP5, Taylor et al. 2012). A consequence of this is that the sea-level projections from the two reports differ only in their estimates of the contribution from Antarctica, for which new ice-sheet modelling results have been incorporated into the SROCC assessment that were not available at the time of AR5.

The difference between the global mean sea-level projections in AR5 and SROCC is negligible under RCP2.6 and RCP4.5. However, the projected global mean sea-level rise under RCP8.5 has been increased in SROCC compared to AR5 by 10 cm at the end of the current century, relative to the baseline period 1986–2005 used in both SROCC and AR5. For global mean sea level, the SROCC projection for 2100 under RCP8.5 is 0.84 m (0.61–1.10 m, *likely* range) compared to 0.74 (0.52–0.98 m, *likely* range) for AR5. This upward revision is in accordance with much of the scientific literature published after AR5 (Golledge et al. 2015; Paolo et al. 2015; DeConto and Pollard 2016). The projected Antarctic contribution to future sea-level rise is, however, still highly uncertain (Yu et al. 2018; Edwards et al. 2019; Golledge et al. 2019), and future revisions may well be sizeable. This is important to keep in mind when such projections are used for coastal spatial planning, since this uncertainty is directly reflected in projected coastal sea-levels around the world.

The primary source of spatial inhomogeneities in sea-level rise for Sweden is GIA, which induces a land rise that varies from less than 1 mm per year in the southernmost part of the country to around 10 mm per year along parts of the Gulf of Bothnia coast (Vestøl et al. 2019). Another process with large potential to drive spatially uneven sea-level rise, particular in strong warming scenarios, is melting of land ice. When ice melts from an ice sheet or glacier, it gives rise to near immediate changes in earth’s gravitational field and rotation, as well as crustal deformation. These changes are specific to the location of the melt source, and they give rise to distinct geographical sea-level change patterns known as fingerprints (Plag and Jüttner 2001; Kopp et al. 2010; Brunnabend et al. 2015). Generally speaking, a sea-level fall is induced in the vicinity of the melt source, and sea-level rise in the far field. For Sweden specifically, this means that melting a given mass of ice on Antarctica gives a much larger sea-level rise than if the same ice mass was melted on Greenland. This large difference in the susceptibility to melt from the two polar ice caps is also investigated here using a new kernel approach to sea-level fingerprints introduced by Mitrovica et al. (2018).

Lastly, the impact that these new sea-level rise projections have on the frequency of sea-level extremes is also quantified and discussed in some detail. In particular, we quantify how the return period of a given sea-level extreme changes as a function of time owing to mean sea-level change under the different scenarios RCP2.6, RCP4.5 and RCP8.5. An effort is also put into discussing the considerable uncertainties that are inherent in estimates of both future mean and extreme sea levels.

## Materials and methods

### Mean sea-level projections & GIA

**Fig. 1:**
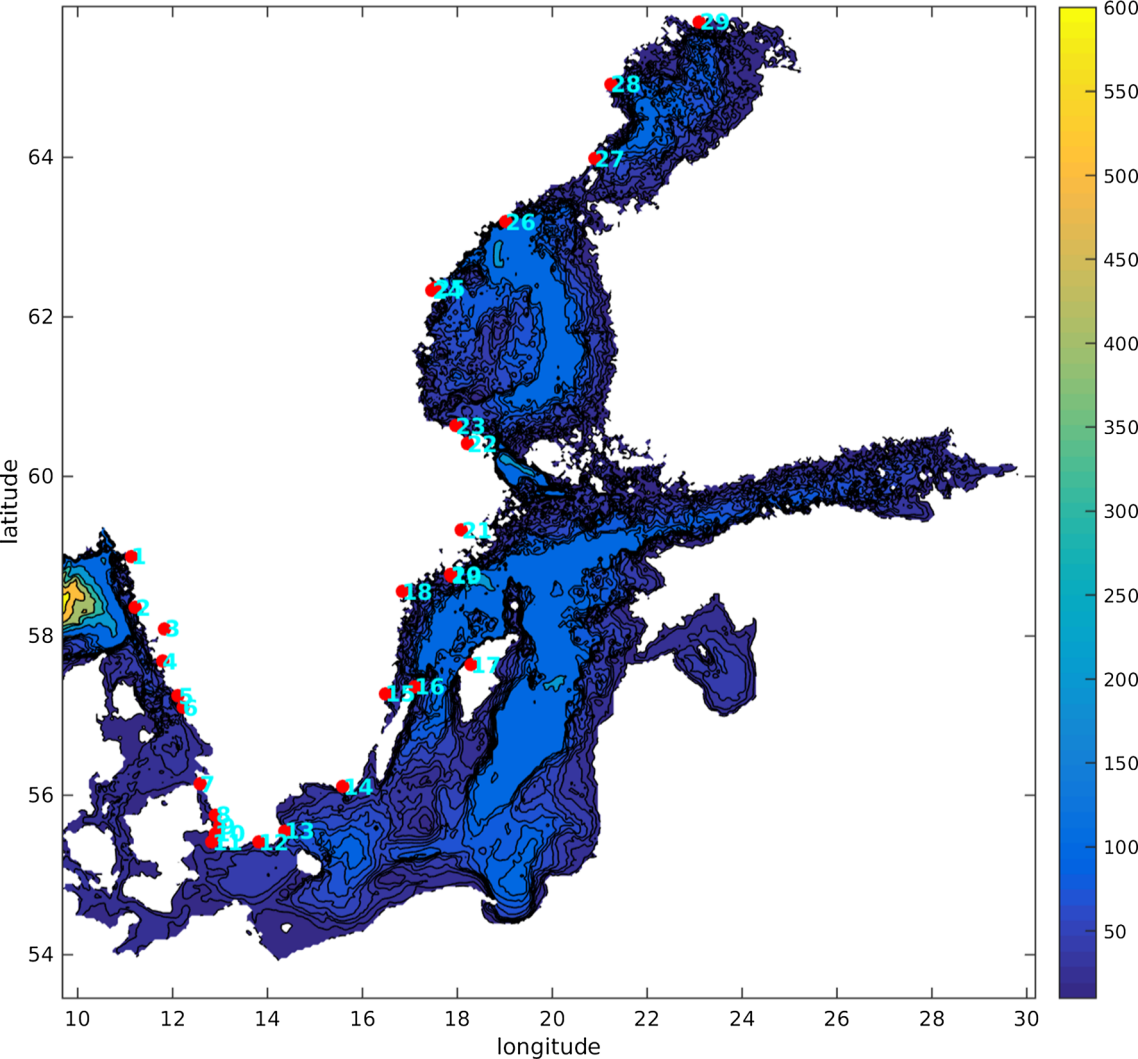
Bathymetric chart showing the locations of the tide gauge stations. Depths are given in meters; station names and further info on the stations are given in Table 1

Our mean sea-level projections are presented at 29 tide gauge stations located around the Swedish coast. The locations of the stations are shown in Fig. 1 and station names and further info on the stations are given in Table 1. The mean sea-level change at the different stations is interpolated, using Matlab’s scattered interpolant routine, from the spatially varying fields in SROCC, which have a spatial resolution of one degree. The yearly mean sea-level rise time series at each station is then fitted to a third-degree polynomial, to smooth out interannual variability. Our projections show the ensemble median for each RCP. The one-degree spatial resolution is much too coarse to give good estimates of dynamic sea-level changes induced by ocean circulation changes in Swedish coastal waters. This is particularly true for the Baltic Sea, which is represented by only a handful of gridboxes at that resolution. The steric and dynamic sea-level changes that are captured in these projections are thus occurring on larger spatial scales than those of the Baltic Sea. The projected GIA is taken from high-resolution measurements instead of those used in SROCC and AR5. The melting fingerprints for Greenland and Antarctica both have spatial scales that are reasonably well resolved at a resolution of one degree.

**Table 1:** Information from the different stations. Number of statistical years is the number of years with data coverage exceeding 80% that we included in the GEV analysis. The first number alludes to years starting in July and ending in June; the value in brackets alludes to years counted from the day the station was first installed

Station number	Station name	Years available	Number of statistical years
1	Kungsvik	1973–2017	42 (43)
2	Smögen	1910–2017	106 (106)
3	Stenungsund	1962–2017	50 (50)
4	Göteborg-Torshamnen	1967–2017	48 (49)
5	Ringhals	1967–2017	46 (47)
6	Varberg	1886–1982	85 (89)
7	Viken	1976–2017	40 (41)
8	Barsebäck	1982–2017	24 (24)
9	Malmö	1924–1963	35 (37)
10	Klagshamn	1929–2017	81 (83)
11	Skanör	1992–2017	24 (25)
12	Ystad	1886–1987	99 (100)
13	Simrishamn	1982–2017	34 (34)
14	Kungsholmsfort	1886–2017	129 (130)
15	Oskarshamn	1960–2017	55 (56)
16	Ölands norra udde	1961–2017	50 (51)
17	Visby	1960–2017	55 (56)
18	Marviken	1964–2017	51 (52)
19	Landsort	1886–2006	119 (120)
20	Landsort Norra	2004–2017	11 (12)
21	Stockholm	1889–2017	127 (128)
22	Forsmark	1975–2017	40 (41)
23	Björn	1891–1978	82 (82)
24	Draghällan	1897–1969	66 (66)
25	Spikarna	1968–2017	47 (48)
26	Skagsudde	1982–2017	23 (24)
27	Ratan	1891–2017	124 (125)
28	Furuögrund	1916–2017	100 (101)
29	Kalix	1974–2017	41 (41)

Our GIA estimate is taken from the semi-empirical NKG2016LU model (Vestøl et al. 2019) that combines a GPS measurement-based empirical model and a geophysical glacial isostatic adjustment model. The NKG2016LU model gives the most recent and accurate estimates available for the current post glacial rebound in Sweden, and we have assumed that the current rate of land rise is unchanged during the period 2019–2100. A direct comparison between NKG2016LU and values interpolated from the one-degree GIA fields used in SROCC and AR5 shows differences in landrise between 2019 and 2100 exceeding 10 cm for some stations in northern Sweden. High-resolution GIA is thus a key component for these projections.

For the mean sea-level rise, we also calculate a time of emergence (TOE) (Hawkins and Sutton 2012). TOE is a subjective but often used metric that indicates when a forced signal becomes sizeable compared to natural variability. In this study, we define the TOE as the year when the mean sea-level change at a given station is twice as large as the standard deviation of that stations yearly mean sea level, which is calculated from linearly detrended tide gauge data. The metric is thus dependent both on the sea-level variability at the station and the sea-level projection, and all stations will not necessarily experience a TOE within the time frame of the projections. Moreover, the shortness of the records on some of the stations implies that the calculated standard deviation of the yearly mean sea level can be influenced by multidecadal variability. However, calculating the standard deviation of the yearly mean sea level using 23-year periods extracted from the long record at Kungsholmsfort shows that the standard deviation calculated for different periods differ by less than 2 cm, so this effect is likely small when the mean sea-level rise is sizeable.

### Fingerprints

The sea-level fingerprints we use to calculate the mean sea-level rise are taken from SROCC as part of the interpolations of the Antarctic and Greenland contributions to sea-level rise. However, Mitrovica et al. (2018) recently published a set of fingerprints, where individual sea-level kernels are available for almost all our tide gauge stations. We use these new kernels in Sect. 3.2 to look at the sensitivity to the ice sheet melt geometry for the individual stations. These kernels are defined through

1 $$\begin{aligned} SL(r_0)=\int \int _\varOmega \rho _I I(\theta ,\phi )K_0(\theta ,\phi )d\varOmega \end{aligned}$$

where $SL(r_0)$ is the sea level at the site $r_0$, $\rho _I$ is the density of ice, *I* is the change in ice height, $K_0$ is the sensitivity kernel for the site $r_0$ (in units of ${{\text{mkg}}^{-1}}$), $\theta$ and $\phi$ are the colatitude and longitude and the integration is performed over the surface of the earth. The information encoded in the kernels is thus a map of how the sea level at the given site, $r_0$, is affected by melt from different localities on Antarctica and Greenland. These kernels thus give us a way of investigating the right-hand side of Eq. (1), while the fingerprints we use from SROCC only give us the left-hand side. Not having either SROCC kernels or the time resolved melt geometry used to compute the SROCC fingerprint, we cannot estimate the difference between the two fingerprint products. However, the kernel means we calculate are in quite good agreement with the Baltic Sea fingerprints shown by (Grinsted 2015).

The statistical properties of the kernels are examined in Sect. 3.2. Together with means and standard deviations, we also look at the skewness and kurtosis of the distribution of kernel values on spatial points in Greenland and Antarctica. Skewness is a measure of the asymmetry of a distribution. The kurtosis of the normal distribution is 3, and a value higher than this indicates that the distribution produces more and more extreme outliers than the normal distribution, while the opposite is true if the kurtosis is less than 3. These higher moments of the distributions are defined according to,

2 $$\begin{aligned} {\text{Skew}}(x)= & {} \frac{E\left[ (x-\mu )^3\right] }{(E\left[ (x-\mu )^2\right] )^{3/2}} \end{aligned}$$

3 $$\begin{aligned} {\text{Kurt}}(x)= & {} \frac{E\left[ (x-\mu )^4\right] }{(E\left[ (x-\mu )^2\right] )^2} \end{aligned}$$

where *E* is the expected value and $\mu$ is the mean value.

### Sea-level extremes

Our analysis of sea-level extremes is based on hourly data from the 29 tide gauge stations. All station data between when the station was first installed and February of 2017  or when the station was discontinued are used in the statistical analysis. The tide gauge data are linearly detrended to remove the mean sea-level change from the time series. Extreme sea-level statistics such as return levels and return periods are found from fitting generalized extreme value (GEV) distributions to time series of annual maximum sea level from the different stations (Coles 2001). The goodness of fit was evaluated using a chi-squared test. The test rejected the null hypothesis that the observed set of yearly sea-level maximum was drawn from the fitted GEV distribution at the 0.05 level at three stations: Furuögrund, Kalix and Kungsholmsfort. The empirical and fitted cumulative distribution functions (CDFs) from those stations are shown and discussed in the Supplementary Material S1. Visually, these fits do not look particularly bad, especially not for the high percentiles that are used to calculate the return levels, so we have kept also those stations in the analysis.

A return level is defined as the highest sea level we expect to see within a return period. That is, a 100-year return level is a sea level typically seen only once in a 100-year period. Since sea-level extremes are more frequent in winter, we use a year starting in July and ending in June for this analysis. We also somewhat arbitrarily impose the threshold that each year we use in the analysis should have at least 80% data availability. The length of the observational time series differ a lot between the stations, and for many stations, they are clearly too short to be used to reliably estimate the 200-year return levels that we calculate in Sect. 3.3. However, short records are the only records available in many places, and for demonstrational purposes, we have chosen to do the same calculations at all stations regardless of the length of the time series. Uncertainty owing to short records and other factors is discussed in the Sect. 4.1

## Results

### Mean sea-level change

**Fig. 2:**
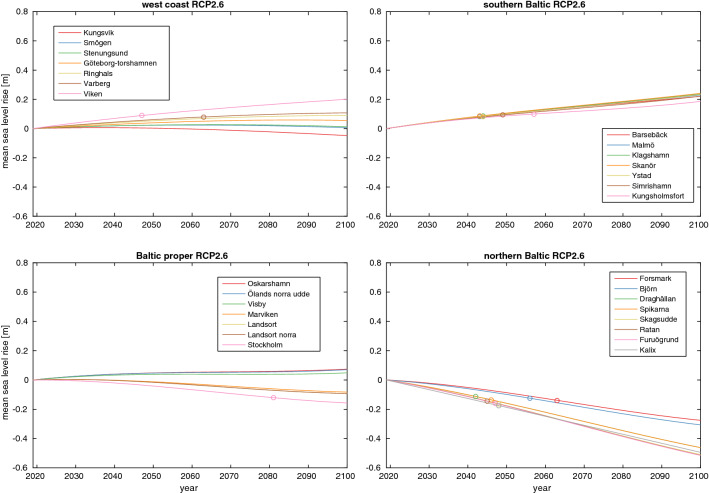
Mean sea-level change at the different stations in the RCP2.6 scenario. All components of sea-level rise is from SROCC except GIA, which is from the NKG2016LU model. The station are sorted into four geographical areas: the west coast, the southern Baltic, Baltic proper and northern Baltic. TOE is marked with rings

**Fig. 3:**
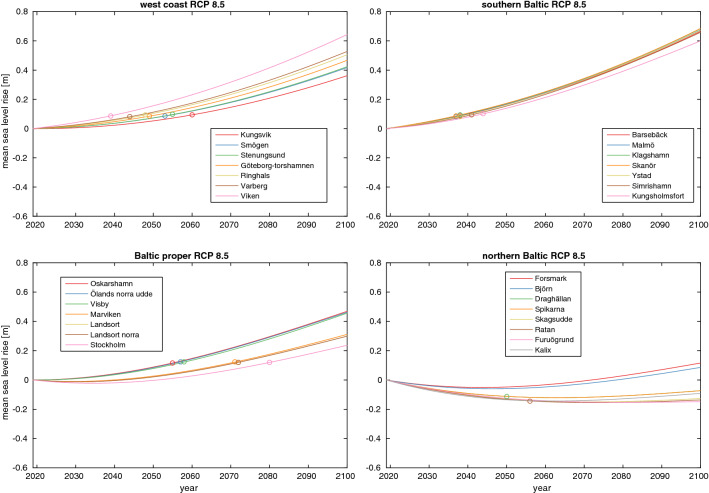
Same as 2, but for the RCP8.5 scenario

Figures 2 and 3 show the mean sea-level rise for RCP2.6 and RCP8.5 at the tide gauge stations; the same figure for RCP4.5 is found in Fig. S2. The stations are split into four different areas: the west coast, the southern Baltic, Baltic proper and northern Baltic. The southern Baltic is clearly the most vulnerable to sea-level rise, while the northern Baltic is the least vulnerable. The key difference is GIA, which varies from about 10 mm/year at some locations in the northern Baltic to less than 1mm/year in the southern. The southern Baltic stations are also those most exposed to sea-level rise from melting on Greenland, which is further discussed in Sect. 3.2. This is also a primary reason for why the scenario difference is larger at the southern Baltic stations than at the northern ones. The average difference between the RCP2.6 and RCP8.5 projections at the southern Baltic stations is 44 cm, while this difference is 39 cm at the northern Baltic stations.

The usage of regional estimates for all terms instead of just for GIA as was done by Nerheim et al. (2018) results in a lower projected sea-level rise at the end of the century. This methodological difference gives rise to projection differences that exceeds 5 cm at many stations and 10 cm at some stations in the RCP8.5 scenario in 2100. The regional sea-level rise in Sweden is generally lower than the global mean sea-level rise that was used in Nerheim et al. (2018), and the primary cause of this is that Sweden gets a smaller than average sea-level rise contribution from melting on Greenland. The difference may not seem particularly large, but it is similar in magnitude to the difference in mean sea-level rise in 2100 between the RCP2.6 and the RCP4.5 scenario. It is also roughly equal to the difference between the AR5 and SROCC projections of sea-level rise in the year 2100 under RCP8.5. Thus, our more careful treatment of spatial inhomogeneities has lowered the projected sea-level rise for Sweden by about as much as the larger Antarctic contribution has added to it, and the new projections are thus still broadly consistent with those of Nerheim et al. (2018).

TOE, a measure of the time when the mean sea-level change is sizeable compared to the natural variability (see Sect. 2.1), is shown with rings in Figs. 2 and 3 for the different stations. Under RCP2.6 it is only in the southern and northern Baltic where all stations experience a TOE. At both locations, it happens around mid-century, and at the northern stations, the TOE is a consequence of sea-level fall. In the RCP8.5 projection, all stations, except most of those in the northern Baltic, experience a TOE, and this occurs before 2040 at most of the southern Baltic stations. A number of stations on the west coast and in the Baltic proper first see a rise and then a drop in sea level under RCP2.6, while the opposite order of events occurs at some stations in the Baltic proper and the northern Baltic under RCP8.5. This behaviour is caused by a competition between an accelerating or decelerating sea-level rise, and a constant land rise. A TOE could, in principle, occur more than once when the mean sea-level trend changes sign, however, we don’t see that happening this century at any of our stations.

Even in the RCP8.5 projection, most northern Baltic stations have experienced a sea-level drop between 2019 and 2100. However, it is also clear from Fig. 3 that the rate of change of sea-level at those northern Baltic stations is either positive or close to zero at the end of the century. GIA is thus not large enough to induce much sea-level fall after 2100 in the RCP8.5 projection.

### Fingerprints: A great Greenland–Antarctica contrast

The spatial variability of the projected sea-level rise at the stations is dominated by GIA, which is ongoing since the last ice age. Apart from GIA, the largest potential for spatially inhomogeneous sea-level rise comes from land ice melt through the sea-level fingerprints. To look at the sensitivity to melt geometries on Greenland and Antarctica for the different stations, we use the station-based sea-level kernels of Mitrovica et al. (2018), which are discussed in more detail in Sect. 2.2. Simply put, the kernels are two dimensional fields, defined over earth’s major ice fields, that show how the sea level at a given station is influenced by melt from different locations on these ice fields.

**Fig. 4:**
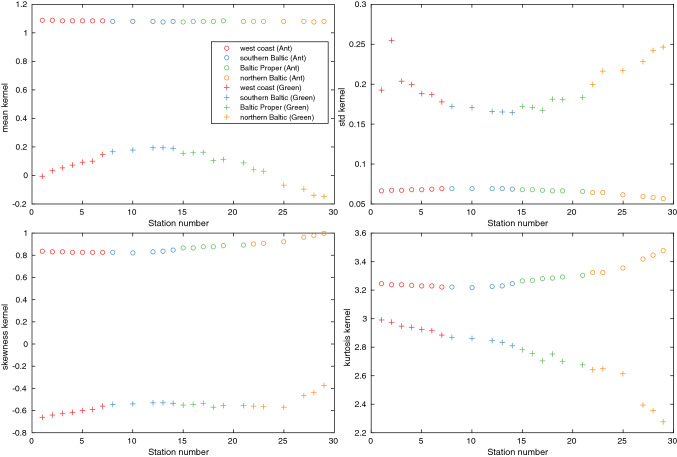
Mean, standard deviation, skewness and kurtosis of Antarctic and Greenland sea-level kernels. The kernel values are normalized so that a kernel value of 1 signifies that melting a kilo of ice has approximately the same sea-level effect as adding a kilo of water with density 1030 ${{\text{kgm}}^{-3}}$ to the global ocean would if the water was distributed uniformly. The statistics for Greenland (Antarctica) are evaluated over all ice-covered grid points on Greenland (Antarctica). The geographical areas are the same as in Fig. 2

For simplicity, we have here grouped together the 2D kernel fields geographically into one Antarctic and one Greenland set for each station. The kernels are also normalized so that a value of 1 signifies that the sea-level rise caused by melting a kilo of ice is equal to the sea-level rise caused by uniformly distributing a kilo of water with density 1030 ${{\text{kgm}}^{-3}}$ over the global ocean. Statistics from the kernel sets for Greenland and Antarctica for our different stations is shown in Fig. 4. The kernel means are much smaller and the spatial variability is much greater for Greenland than for Antarctica. This is owing to Sweden’s relative closeness to Greenland. The response to melt on an ice sheet is typically a sea-level fall within 2000 km of the area where the melt occurs, due to both a reduced gravitational pull by the ice sheet and elastic uplift of the crust in response to the ice unloading and sea-level rise in the far field (Mitrovica et al. 2018). Melting on Eastern Greenland would consequently lead to a sea-level drop in parts of Sweden, while regardless of where it occurs all Antarctic melt will lead to sea-level rise.

The vulnerability to Antarctic melt is almost uniform for the Swedish coast. However, the Southern Baltic expects about 20% of the globally averaged sea-level rise from melting on Greenland, while the northern Baltic expects a sea-level fall, so the response to melting on Greenland is highly uneven. The higher moments of the distributions are also different. The Antarctic kernels are right skewed and those for Greenland are left skewed. Moreover, all kernel distributions for Antarctica have positive excess kurtosis, while all those for Greenland have negative excess kurtosis. Thus, outlier are more (less) frequent and/or more (less) extreme for the Antarctic (Greenland) distributions than they would have been if those were normally distributed. The interpretation of these higher moments for Antarctica is that both the right skew and the excess kurtosis suggest that certain Antarctic melt geometries can give greater sea-level rise in Sweden than would have been possible if the kernel values were normally distributed. However, given the low standard deviation of the Antarctic values, this is not much of problem, and the sea-level rise at the Swedish coast can to a fair approximation be taken as independent of where on Antarctica the melt occurs. For Greenland, the situation is quite different, here the skewness and kurtosis instead suggest that the distributions are generally light-tailed, but with more weight on its left side. The left tail, of course, hold the values from locations where ice melt gives the smallest sea-level rise or the greatest sea-level fall. This corresponds to the eastern part of Greenland, which is closest to Sweden. Currently, the largest mass loss from Greenland occurs in the western, north western and south eastern parts (Mouginot et al. 2019). However, the largest potential for future sea-level rise comes from the northern and north eastern parts of the island (Mouginot et al. 2019). These areas have mostly intermediate kernel values. Melting on Greenland is thus expected to have only a minor influence on future sea-level change in Sweden. In conclusion, the overall uncertainty in future sea-level rise for Sweden is not strongly dependent on the projected melt geometries. For Antarctic melt, this is because the standard deviation of the kernels is small. For melt on Greenland, it is because the kernel means are small and the kernel distributions are left skewed and light-tailed.

### Extreme sea-level changes

**Fig. 5:**
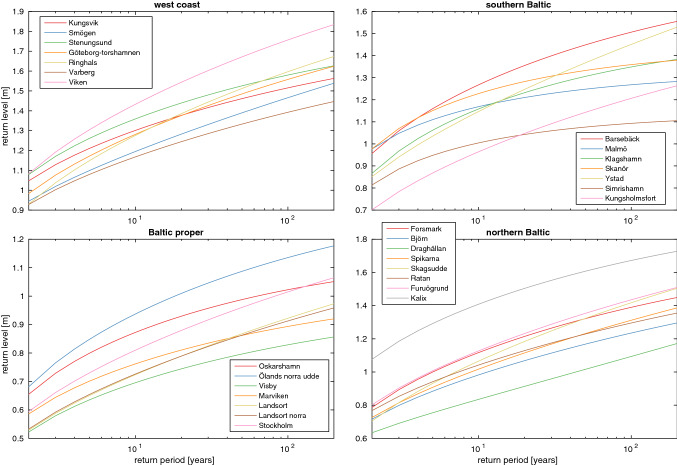
Return level as a function of return period for the different stations. The geographical areas are the same as in Fig. 2. The *x*-axis is logarithmic

The return level as function of the return period for the different stations is shown in Fig. 5. A return level is the level the water is expected to reach on average only once in a return period, and it is an often used statistic, for example, when planning construction in coastal areas. The return levels are interesting for such purposes because they suggest how likely it is that a storm flood will reach a given structure. Climate change, however, severely complicates this seemingly simple relationship in two important ways. The first way has the greatest potential to achieve large changes and is a direct effect of changing the mean sea level. Figure 5 suggests that the difference between the height of a 100- and a 200-year return level is typically about 10 cm at our stations, and a mean sea-level increase of 10 cm would thus imply that the current 200-year return level should be expected to occur instead every 100 years. The second way is due to climate change affecting the storminess of the region, which can be very important locally. How storm surges and the wave climate along the Swedish coasts could be affected by meteorological changes is highly uncertain and we have not tried to quantify such changes here. The interested reader is refereed to Nikulin et al. (2011), Hieronymus et al. (2017, 2018) and Mentaschi et al. (2017) for some discussions of the effects of changing winds, waves and water depth on sea-level extremes.

The effect of a given mean sea-level change on the return period for the current 100-year return level is illustrated in Fig. S3. Since large parts of the Swedish coastline will experience sea-level fall in most scenarios, we also probe the effect of a sea-level fall. Return levels are monotonically increasing and concave functions of the return period. Even a modest sea-level fall can therefore lead to large changes in the return period of a given return level. A consequence of this is that a sea-level fall of 10 cm is enough to give the current 100-year return level, a return period of more than 200 years at all stations. There is more spread in the response to sea-level rise, but a sea-level rise of 40 cm would make the return period of the current 100-year return level less than 10 years at all stations.

**Fig. 6:**
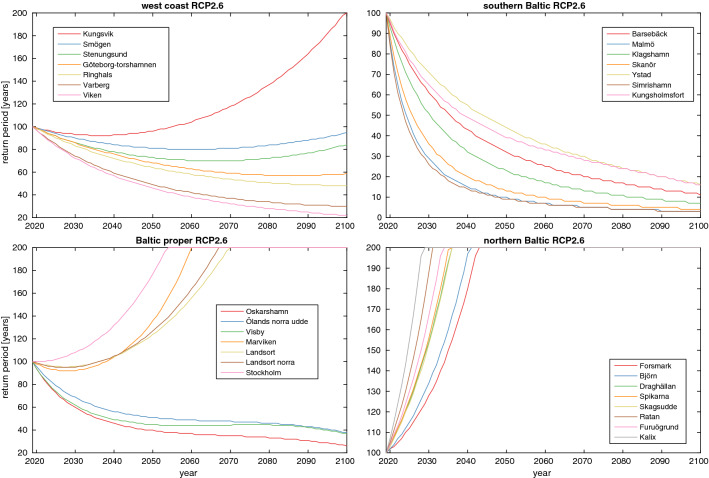
Return period of the current 100-year return level as a function of time following RCP2.6 at the different stations. The geographical areas are the same as in Fig. 2

**Fig. 7:**
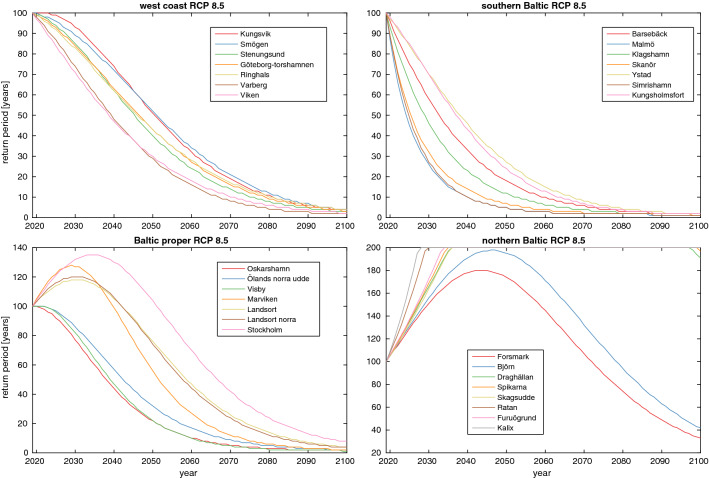
Return period of the current 100-year return level as a function of time following RCP8.5 at the different stations. The geographical areas are the same as in Fig. 2

While it is interesting to know the sensitivity of return periods to sea-level rise, it is also hard to base coastal planning on such data without knowledge of when a certain sea level may be reached. To remedy this problem, we have calculated the change in return period of the current 100-year return level as a function of time in RCP2.6 and RCP8.5, which is shown in Figs. 6 and 7. The corresponding figure for RCP4.5 is shown in Fig. S4. The figures are made by combining the information in Figs. 2 and 3 with that in Fig. S3. The return period will of course increase with time at stations where the sea-level falls and vice versa. The temporal development differs strongly between stations and scenarios, but the southern Baltic is always the most vulnerable area. Here, we find that even in RCP2.6 we have a return period of less than 20 years for the current 100-year return level at the end of the century, while the same is true already in 2060 in RCP8.5. An inescapable conclusion that can be drawn from Figs. 6 and 7 is thus that high sea levels that occur seldom today in the Southern Baltic will be relatively commonplace towards the end of the century regardless of which climate scenario we follow. Moreover, the current 100-year return level in the Southern Baltic will be reached nearly every year after 2100  under RCP8.5, and more often than every ten years under RCP4.5 (see Fig. S4).

## Discussion

### Uncertainties

Very large uncertainties pervade both the projected future mean sea levels and the estimated return levels. The uncertainty in future mean sea levels can be decomposed into components owing to internal climate variability, model uncertainty and scenario uncertainty. The overall uncertainty tends to be more dominated by scenario uncertainty the longer the projections get (Hawking and Sutton 2009). From a process-based point of view, it is the uncertainty in the future sea-level contribution from Antarctica that tends to dominate the overall uncertainty (Kopp et al. 2014, 2019; Oppenheimer et al. 2019). The scenario uncertainty alone gives a range of mean sea-level rise in the year 2100 at the most vulnerable southern Baltic stations that cover values between about 20 and 70 cm. To this, we can add, at least, a *likely* range that adds 26 cm to the global mean projection under RCP8.5. The *likely* range, however, is not an upper bound on the potential for sea-level rise, and the SROCC report mentions that a mean sea-level rise exceeding 2 m in 2100 cannot be completely ruled out. However, the probability of such an outcome under a given RCP is currently not well constrained. Moreover, the uncertainty range has grown in the last few IPCC reports, and since so much of it is owing to the choice of scenario, we will undoubtedly have to live with deep uncertainty in future sea-levels also in the coming years. That is, even if we could produce perfect models (i.e. having a *likely* range that adds 0 to the mean estimate), we would still have a very considerable spread depending on which emission scenario our future society will follow.

The uncertainty in the estimated return levels is thought to be somewhat smaller than that in future mean sea-level rise at least in the longer perspective, owing to the large potential for mean sea-level rise from melting ice sheets. However, the uncertainty in estimated return levels is clearly also important and it comes from many different sources. The two most common ways of estimating return levels is to fit either a GEV distribution to a distribution of annual sea-level maxima as we do here or to fit a generalized Pareto distribution to a distribution of sea levels higher than a given threshold. Such methodological differences in the calculation of return levels can give substantially different estimates (Wahl et al. 2017). Moreover, likelihood based 83% confidence bounds on the GEV fitted return levels, here chosen to give a range similar to the *likely* range as defined in the IPCC reports, typically adds an uncertainty of around 10 cm to the estimated 100-year return levels.

However, the main and most problematic uncertainty is owing to the lengths of the observational records at the stations. Inferring a 200-year return level from an 11-year observational record, as we do in for Landsort Norra, is obviously not good practice. Nevertheless, in this case, it actually appears to work out well, since the return level is very similar to that calculated from the much longer record at the nearby station Landsort. The uncertainty owing to short records is, much like the uncertainty in future greenhouse gas emissions, hard to reduce. Earlier work has shown that extreme value statistics can be highly sensitive to outliers. Dangendorf et al. (2016), for example, found that the estimates of the 200-year return level went up by almost 40 cm in parts of the southwestern German North Sea coastline, when the record breaking water levels of the storm Xaver was included in the estimate. Similarly, we found an even stronger influence at Skanör by the choice of how to define a year in the annual maximum times series. All return levels so far have been calculated using a year starting in July and ending in June. However, in Fig. S5, we test to instead start our year whenever the measurement time series starts, while still keeping the 80% data availability threshold. The difference between Figs. 5 and S5 is small for most stations, but for the 200-year return level at Skanör, it is about 50 cm. The choice of data availability threshold together with the definition of year has thus clearly excluded, at least, one very powerful storm from our original estimate, and it strongly affected the statistics. An inescapable conclusion from our investigation and from that of Dangendorf et al. (2016) is thus that rules of thumb of the kind “the X-year return level can be accurately estimated from Y years of data” are destined to fail. Moreover, there is good data supporting that sea levels reached during a storm in the Southern Baltic in 1872 have multi-millennial return periods when inferred from extreme value statistics based on tide gauge records. Such long return times are likely unrealistic, and at least for the Southern Baltic Sea, it seems that extreme value statistics underestimate the probability of these rare events (Fredriksson et al. 2016).

Another problem with the relative shortness of the observational sea-level archives is that they are often too short to accurately reflect the natural variability on decadal to centennial timescales. Lang and Mikolajweicz (2019) found from a 1000-year integration of an ocean model that the 100-year return level in the German Bight as determined from different 100-year time series could differ by more than 1 m. These large variations are probably partly due to a few extreme storms, but they are also in part owing to internal variability on centennial timescales. In our case, one could perhaps hope that data from the large number of stations available could somehow be combined to compensate for the, in comparison with a 1000-year integration, relative shortness of the records at each station. This is, however, not the case since the annual maximum time series are highly correlated at different stations and thus reflect the same climate variability. For example, the average correlation coefficient between the annual max sea-level time series at the west coast stations is $r=.76$, while that at all the Baltic stations is $r=.61$. The correlation between the annual maximum sea-level time series at the west coast and the northern Baltic stations is weaker, averaging $r=.40$.

The underlying reason for the strong correlation between the annual maximum time series at the different stations is that the area our stations cover is small enough to be affected by roughly the same large-scale climate variability. Several different atmospheric patterns, such as the North Atlantic Oscillation (NAO), the East Atlantic Pattern (EAP) and the Scandinavian Pattern (SCA), have previously been implicated in the variability in mean sea levels and storminess in the North Sea and Baltic Sea areas (Andersson 2002; Seierstad et al. 2007; Chafik et al. 2017). The connection between these patterns and extreme sea levels is less explored than that for the mean sea level, although some work exists (see, e.g. Lang and Mikolajweicz (2019) for an investigation in the German Bight).

**Fig. 8:**
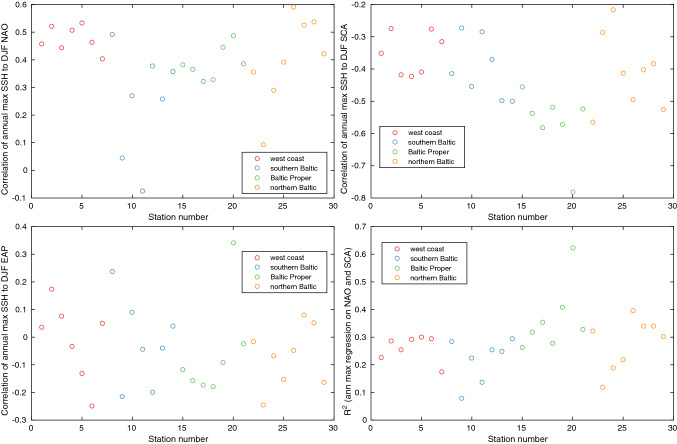
Pearson correlation coefficients and *R*-squared between annual maxima sea-level time series and the NAO, SCA and EAP indices. Correlations and regressions are calculated using the full number of years available at the stations, meaning that the uncertainty in these calculations vary between stations. Data sources for the different indices are given in the acknowledgement

Figure 8 shows the correlation between the annual maximum sea-level time series at the stations and the December–January–February (DJF) averages of the climate indices time series. Most stations show a consistent association between their yearly maximum sea-level time series and the NAO and SCA indices, while the correlation to the EAP index is weak. The regression of the annual maximum sea level onto the DJF SCA and NAO indices show that on average, about 30% of the variance in the annual maximum time series can be explained by the variations in these indices. The highest value of over 60% explained variance is found for the station Landsort Norra, which has the shortest time series of all the stations. Given the proximity of Landsort Norra to Landsort, we expect the explained variance at Landsort Norra to approach that at Landsort as the time series gets longer.

### Coastal spatial planning

Swedish municipalities enjoy a planning monopoly for land within the municipality, so the role of expert government agencies such as SMHI is not to make decisions on coastal spatial planning. Rather, SMHIs role is to support such planning with meteorological, hydrological, climatological and oceanographic expertise, and it is in this light these new sea-level projections should be viewed. Therefore, we as authors give no recommendations on which emission scenario or return-level municipalities should plan for. However, we do support that the new SROCC projection should be adopted in place of the old ones from AR5, since considerable progress has been made on the sea-level problem since AR5. Moreover, climate projections are just one of the factors going into decisions on coastal spatial planning. That is, while knowing the risk that a certain structure will be flooded in 2100 under, for example, RCP4.5 certainly helps deciding whether it is worth building, it is also clearly not enough information to base such a decision on. Further information such as, for example, the economic and societal consequences of a potential flooding must also be taken into account.

Decision-making on coastal spatial planning is thus a complex undertaking involving expertise from several different fields (Hinkel et al. 2018). We find that this complexity is often underestimated and consequently that the answer to the question like where one should or should not build is often simply given in terms of the projected sea-level rise in the year 2100, complemented with estimates of storm surge variations at the site considered. We do not tackle socio-economic consequences here, but our Figs. 6 and 7 are aimed to give at least some more nuance to the already complex climate picture by showing how the return period of a chosen return-level changes with time in the different projections. We believe that such illustrations can be a first step to better include the temporal dimension into coastal spatial planning. This dimension has hitherto, at least in Sweden, been almost exclusively left out, and focus has been put almost single mindedly on projected conditions for the year 2100. Moreover, we also think that framing the problem in terms of time can be useful when choosing what uncertainty range to plan for. Say, for example, that the *likely* range adds 20 cm to the sea-level rise projection at a given station in 2100, and that the yearly sea-level rise at that station is then 1 cm per year. Then, one may think of the *likely* range either as an 83th percentile of the stations projected sea-level rise in 2100, or roughly as a 40-year span centred around 2100 when the projected sea level for 2100 is *likely* to be reached. The latter view seems to us to be much more informative on key questions such as when coastal defences need to be built or upgraded.

## Conclusions

The more complete treatment of spatial inhomogeneities in sea-level rise used here gives a reduction in the projected sea-level rise in Sweden compared with projections by Nerheim et al. (2018). This reduction is a result of Sweden getting a smaller than average sea-level contribution from melt on Greenland. Moreover, using sea-level fingerprint kernels we find, in fact, that a small sea-level fall is the expected response to melt on Greenland for most northern Baltic stations. The difference in sea-level rise from the global mean is largest under RCP8.5, exceeding 10 cm in the year 2100 at some stations. However, the increased Antarctic contribution under RCP8.5 in SROCC compared to AR5 increases the sea-level rise projection by about a similar amount as it is reduced by the better treatment of spatial inhomogeneities. Our RCP8.5 projections are therefore close to those in Nerheim et al. (2018). Moreover, the GIA estimates from the high-resolution NKG2016LU model is found to differ by more than 10 cm from the interpolated SROCC GIA values, in the year 2100 at some northern Baltic stations. High-resolution GIA is thus necessary to accurately project sea-level rise in Sweden.

The projected regional sea-level rise in Sweden has large regional variations. For the least exposed northern Baltic Sea coast, the sea level is projected to be lower in 2100 than today for most stations even under RCP8.5, while a sea-level rise of close to 70 cm is projected for the most exposed southern Baltic station in that scenario. This marked difference is manifested in very different TOE at the different coasts and also in very different expectations for future sea-level extremes. For example, the return period for the current 100-year return level under RCP2.6 is expected to be less than 20 years at all southern Baltic stations in 2100, while it is expected to be in excess of 200 years by 2045 at all northern Baltic stations.

We also briefly reviewed the covariability of sea-level extremes at the different stations. The annual maximum sea-level time series were found to be strongly correlated between both the stations on the west coast and the Baltic Sea stations. Moreover, we found the variability in sea-level extremes to be related to large-scale climate variability, in particular to the NAO and SCA patterns. The variability of those two patterns could, on average, explain about 30% of the variance in the annual maximum sea-level time series at the stations.

## Electronic supplementary material

Below is the link to the electronic supplementary material.

Electronic supplementary material 1 (PDF 245 kb)
