# Supplementary material for: Sea-level rise projections for Sweden based on the new IPCC special report: The ocean and cryosphere in a changing climate
Source: Ambio. 2020 Jan 28;49(10):1587–600. doi: 10.1007/s13280-019-01313-8 (PMC7413947; doi:10.1007/s13280-019-01313-8)
Supplement: Supplementary file 1 — Electronic supplementary material 1 (PDF 245 kb) [file 13280_2019_1313_MOESM1_ESM.pdf]

Ambio

Electronic Supplementary Material
This supplementary material has not been peer reviewed.

Title: *Sea-level rise projections for Sweden based on the new IPCC special report: The ocean and cryosphere in a changing climate*

Authors: Magnus Hieronymus and Ola Kalén

GEV goodness of fit

Figure S1 shows the empirical and fitted CDFs at the three stations where the chi-squared test rejected the null hypothesis that the set of maximum yearly sea level was drawn from the fitted GEV distribution at the 0.05 level. Visually the fits do not look particular bad, especially not for Kungsholmsfort and Furuögrund. Both these stations have very long time series, and it it thus easier to find significant differences in the fitted and empirical distributions for those than for stations with shorter records. There are many plausible reasons for why fitting a distribution to these long time series can be difficult. For example, there is a residual nonlinear trend in the tide gauge data after the linear detrending is done, since climate induced sea level changes are not exactly constant in time. Moreover, instruments for measuring sea level have changed multiple times and the older data have been digitalized, both of these changes can lead to discrepancies in sea level records. However, in general we don't believe that finding a distribution with a good fit is the main problem when estimating the return levels at these stations, rather we believe it is shortness of the records that gives rise to the largest uncertainties.

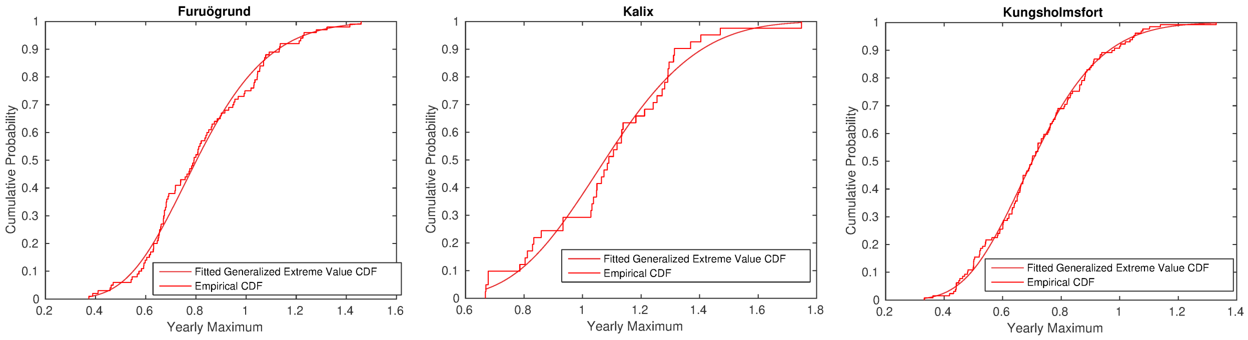

Fig. S1 The empirical and fitted CDFs for Kungsholmsfort, Kalix and Furuögrund

Supplementary figures

The supplementary material presented here shows the projections for RCP4.5, the change in return level for a given mean sea level rise at all stations, and the effect of changing the definition of year when calculating the return levels.

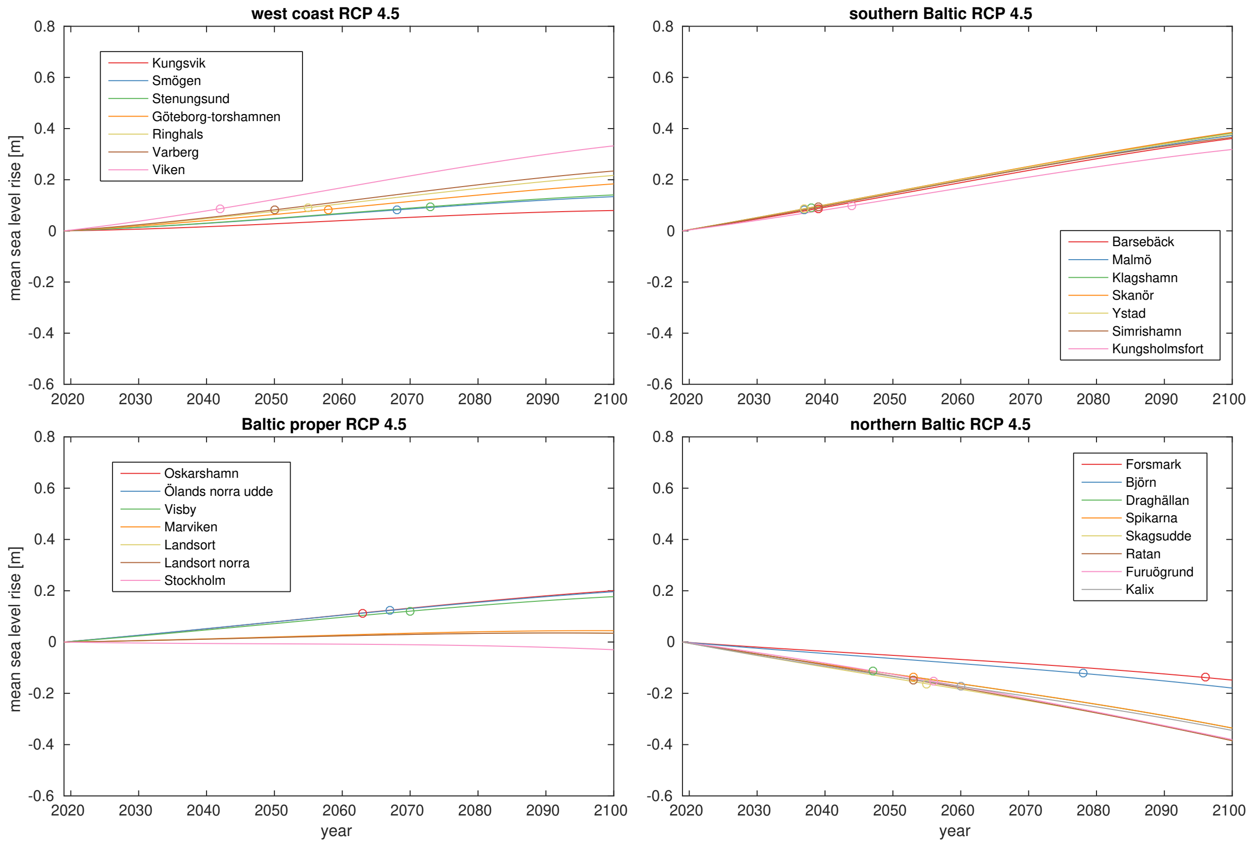

Fig. S2 Same as Fig. 2, but for the RCP 4.5 scenario

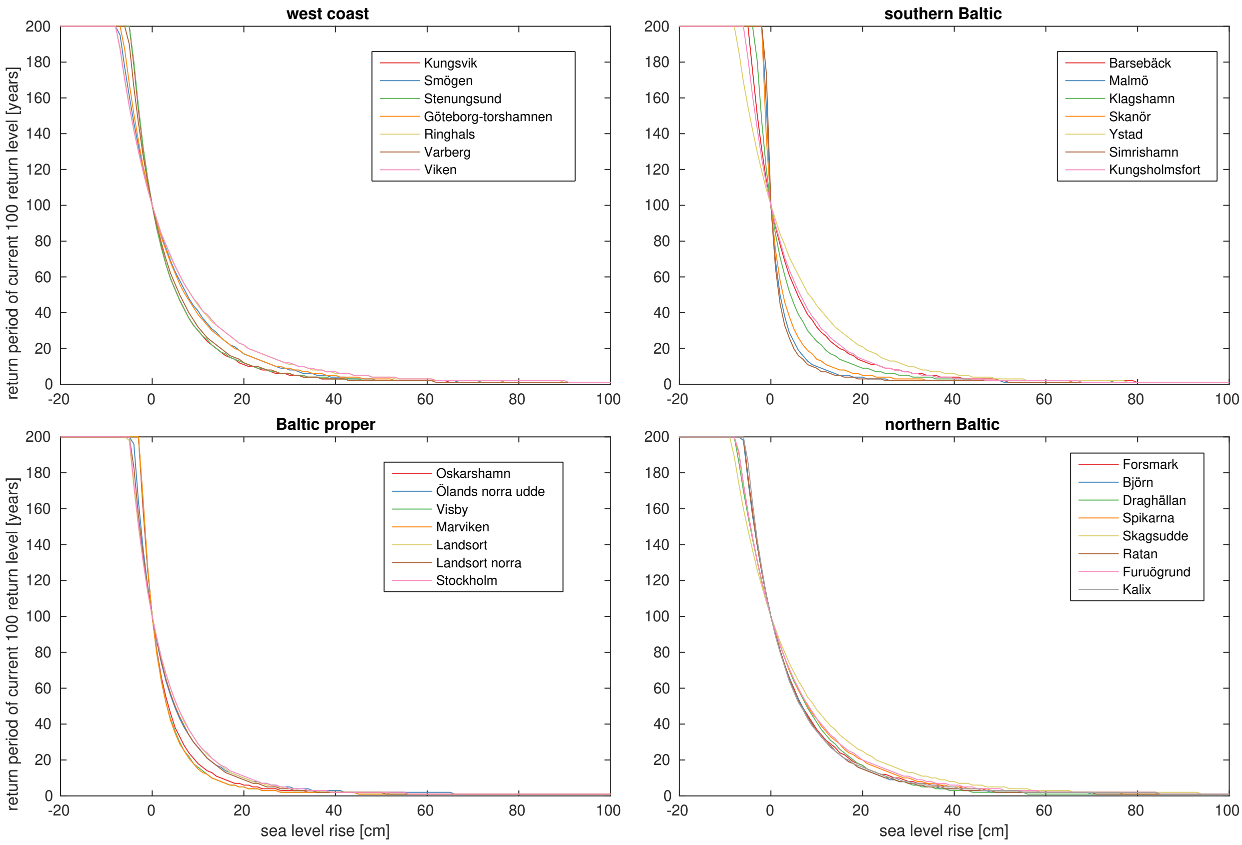

Fig. S3 Return period of the current 100 year return level as a function of mean sea level rise at the different stations. The geographical areas are the same as in Fig. 2.

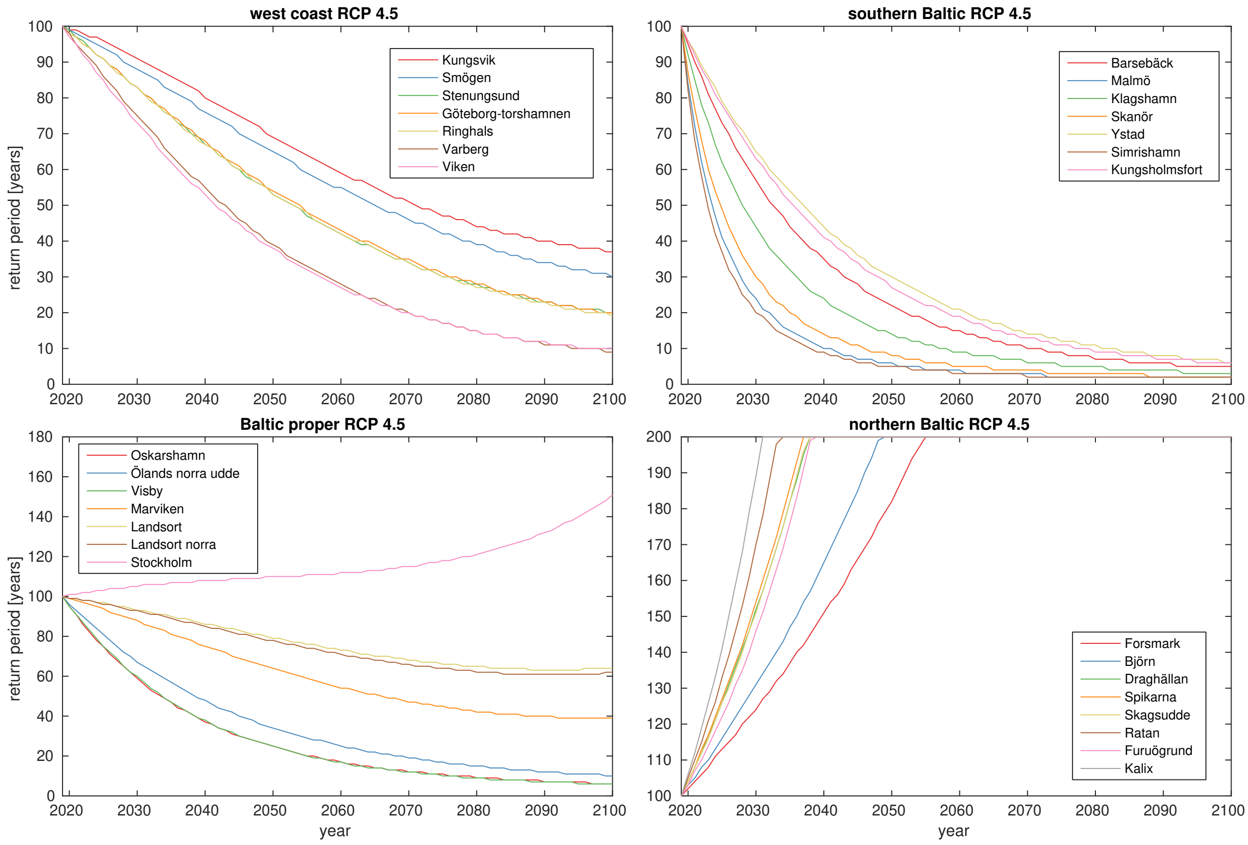

Fig. S4 Return period of the current 100 year return level as a function of time following RCP 4.5 at the different stations. The geographical areas are the same as in Fig. 2.

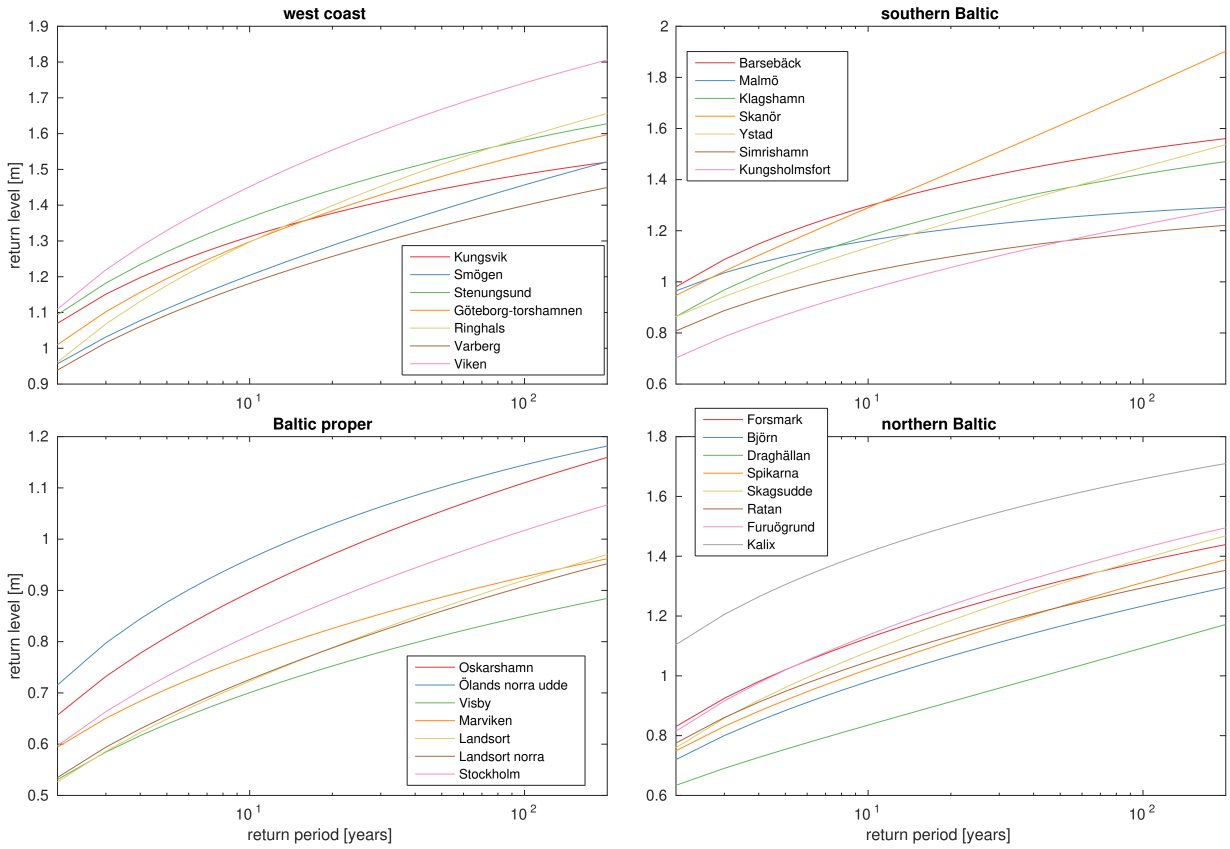

Fig. S5 Same as Fig. 4, but instead of using a year that starts in July we use one that starts whenever the station time series starts.
